# Evaluation of the use of a clinical practice guideline for external apical root resorption among orthodontists

**DOI:** 10.1186/s40510-024-00515-5

**Published:** 2024-04-22

**Authors:** Sebastiaan P. van Doornik, Marlotte B. M. Pijnenburg, Krista I. Janssen, Yijin Ren, Anne Marie Kuijpers-Jagtman

**Affiliations:** 1 Department of Orthodontics, University Medical Center Groningen, Hanzeplein 1, 9713 GZ Groningen, The Netherlands; 2 Center for Dentistry and Oral Hygiene, University of Groningen, University Medical Center Groningen, Antonius Deusinglaan 1, FB 21, 9713 AV Groningen, The Netherlands; 3 Department of Orthodontics and Dentofacial Orthopedics, School of Dental Medicine/Medical Faculty, University of Bern, Hochschulstrasse 4, 3012 Bern, Switzerland; 4 Faculty of Dentistry, Universitas Indonesia, Campus Salemba, Jalan Salemba Raya No. 4, Jakarta, 10430 Indonesia

**Keywords:** Orthodontics, Orthodontic appliances, Fixed, Adverse effects, Root resorption, Practice guideline, Guideline adherence, Evidence based practice, Treatment outcome, Surveys and questionnaires

## Abstract

**Background:**

External apical root resorption (EARR) is a frequently observed adverse event in patients undergoing fixed appliance therapy. Assessing the patients’ risk during treatment is important, as certain factors are assumed to be associated with an increased likelihood of occurrence. However, their predictive value remains limited, making evidence-based clinical decision-making challenging for orthodontists. To address this issue, the Dutch Association of Orthodontists (NvVO) developed a clinical practice guideline (CPG) for EARR in accordance with the AGREE II instrument (Appraisal of Guidelines for Research and Evaluation II) in 2018. The aim of this study is to get insight into the actual utilization and the practical implementation of the guideline among orthodontists. The hypothesis to be tested was that after its introduction, clinical practice for EARR has changed towards the recommendations in the CPG.

**Objective:**

To investigate the use of the 2018 clinical practice guidelines for EARR among orthodontists 3 years after its introduction.

**Methods:**

A questionnaire using a 7-point Likert scale was developed concerning four domains of EARR described in the guideline. The questionnaire was piloted, finalised, and then distributed digitally among Dutch orthodontists. REDCap was used for data collection, starting with an invitation email in June 2021, followed by two reminders. Effect was tested by the Mann–Whitney U test, and the influence of demographic variables was analysed.

**Results:**

Questionnaires were sent out to all 275 and completed by 133 (response rate 48%); N = 59 females and N = 73 males were included; 81% had their training in the Netherlands, 89% had ≥ 6 years of work experience, and 89% worked in private orthodontic practice. One hundred thirty orthodontists (98.5%) reported changes in clinical practice. The biggest positive change in clinical behaviour regarding EARR occurred if EARR was diagnosed during treatment. Sex, clinical experience, country of specialist training, and working environment of the respondents did not affect clinical practices regarding EARR.

**Conclusions:**

This questionnaire demonstrated that, 3 years after introduction of the guideline, orthodontists improved their self-reported clinical practices to a more standardised management of root resorption. None of the demographic predictors had a significant effect on the results.

**Supplementary Information:**

The online version contains supplementary material available at 10.1186/s40510-024-00515-5.

## Introduction

External apical root resorption (EARR) is a prevalent adverse event in patients undergoing orthodontic treatment with fixed appliances [1]. The biological mechanisms underlying EARR are intricate and involve a combination of mechanical, inflammatory, and cellular processes [2]. Orthodontic forces, can trigger an inflammatory response within the periodontal ligament (PDL) and surrounding tissues. This inflammation activates osteoclasts, specialized cells responsible for bone resorption, which can inadvertently target the root surfaces, leading to root resorption. Molecular signals such as RANKL (Receptor Activator of Nuclear Factor Kappa-B Ligand) and osteoprotegerin (OPG) play crucial roles in regulating osteoclastic activity in this context. The PDL is paramount in both periodontal health and EARR. The inflammatory processes associated with EARR can disrupt the integrity of the periodontal ligament, weakening the attachment of teeth to the surrounding bone. Polymorphisms in genes associated with inflammation, bone remodeling, and immune responses have been implicated in the variability observed in EARR outcomes. There are studies that describe associations of the moderate forms of EARR with a total of 16 gene variants, involving the following genes: IL1B, IL1RN, IL1A, IL6, VDR, TNFRSF11B, OPG, RANKL, P2XR7, SPP1, and IRAK1 [2]. These genetic factors may influence the individual's ability to regulate inflammatory responses, osteoclastic activity, and reparative mechanisms.

The severity of root resorption varies and, if undiagnosed, can even result in tooth loss, compromising treatment outcomes [3]. Histologically,  > 90% of all orthodontically moved teeth present with EARR. Clinically, in 48–66% of teeth with EARR, less than 2.5 mm of the original root length is affected, although 1–5% of the cases will experience EARR of more than 4 mm or more than one-third of the original root length [3, 4].

Assessing the patients’ risk during treatment is important, and some factors are assumed to be associated with an increased risk of EARR, including extractions, treatment duration, genetics, sex, and excessive orthodontic force. However, their predictive value remains weak and evidence-based clinical decision-making regarding EARR is difficult for the orthodontist [1–6].

Evidence-based clinical practice guidelines are one of the most reliable and effective tools for improving the quality of care and reducing practice variation between health care providers [7]. The Dutch Association of Orthodontists (NVvO) developed a clinical practice guideline (CPG) for EARR in accordance with the AGREE II instrument (Appraisal of Guidelines for Research and Evaluation II) [8]. The development process for this CPG included a preparation phase, development phase, commentary phase, and authorisation phase. During the preparation phase, in the beginning of 2015, a survey was sent out to all orthodontists in the Netherlands to determine the need for a CPG for clinically relevant EARR, defined as a loss of 2 mm or more of root length. Based on this survey, a task force translated the most relevant issues into four clinical questions [3]. This ultimately resulted in publication of the first Dutch guideline regarding EARR in 2018, summarising current knowledge regarding EARR and providing clinical recommendations. The guideline provided recommendations for the diagnosis of root resorption, patient-related and treatment-related risk factors, a treatment strategy if root resorption is determined to be present during the orthodontic treatment, and clinical follow-up for patients who have EARR at the end of the treatment. However, the actual clinical practices in orthodontic offices 3 years after introduction of the CPG are unknown and, the level of familiarity and implementation of the CPG remains uncharted. Therefore, the aim of this study is to get insight into the actual utilization and the practical implementation of the guideline among its designated users. The study surveyed orthodontists in the Netherlands on the use of the CPG. The hypothesis to be tested was that after its introduction, clinical practice for EARR has changed towards the recommendations in the CPG. The null-hypothesis was that introduction of the CPG has not resulted in any significant changes in clinical practices related to EARR among orthodontists in the Netherlands.

## Materials and methods

### Sample and ethical approval

To evaluate the CPG on EARR introduced in 2018 [3], a survey was conducted 3 years after its publication among all 275 practising orthodontists in the Netherlands registered in the database of the Dutch Association of Orthodontists (NVvO).

All orthodontists practicing in the Netherlands were contacted by e-mail through the secretariat of the specialist association regarding participation in the study. Inclusion criteria were being registered as a dentist and orthodontist in the mandatory Dutch dental register (BIG register) and practising in the Netherlands.

Exclusion criteria were not being clinically active. Postgraduates were also excluded from the study. The orthodontists received an introduction e-mail providing information about the study with a link to the online questionnaire. A reminder was sent 1 month later. Two months after the reminder was sent, telephone contact was made. Responses were collected anonymously.

Respondents provided informed consent and permission for the data collection and future publication.

The Medical Ethics Review Board of the University Medical Center Groningen (METc UMCG) reviewed the research protocol, registered under UMCG RR 202000771 and METc 2021/274, and concluded that this study is not clinical research with human subjects as meant in the Medical Research Involving Human Subjects Act (WMO) and, therefore, WMO approval is not required.

### Questionnaire

The questionnaire consisted of two parts (Additional file 1). Part A consisted of 13 statements, covering the 13 recommendations and four domains of the EARR guidelines: diagnosis of root resorption (2 questions), risk factors (2 questions), treatment strategy if root resorption is established during treatment (6 questions), and what to do in patients with root resorption at the end of treatment (3 questions). For each of the statements, the participants were asked to indicate their clinical practice regarding the domains of the recommendations before publication (T0) of the EARR guideline and at the time of completing the questionnaire (T1) using a 7-point Likert scale: 1 = never, 2 = very rarely, 3 = rarely, 4 = neutral, 5 = often, 6 = very often, 7 = always. By summing up the responses, cumulative scores for each domain and the total questionnaire were calculated.

Part B consisted of four questions about the background of the respondents: sex (male, female, other), location of specialist education (the Netherlands or elsewhere), years of clinical experience (0–5 years or > 6 years), and present employment (employment as a practice owner or practice employee; working part-time in a hospital, university, or specialist centre). Multiple answers were possible for type of employment.

The questionnaire underwent an initial pilot testing phase involving nine postgraduates to evaluate factors such as question comprehensibility, ambiguity, questionnaire structure, and completion time. Subsequently, the questionnaire underwent a rigorous expert review conducted by three orthodontists who had no prior involvement or participation in the study. This expert review aimed to further assess the questionnaire's content validity and ensure its appropriateness and relevance in evaluating the EARR guidelines [9]. The questionnaire was then imported and distributed using the Research Electronic Data Capture (REDCap) program (Indiana University Pervasive Technology Institute, Indiana, USA) hosted on the UMCG network [10, 11].

### Statistical analysis

Statistical analyses were performed using SPSS Statistics for Windows (Version 26.0. Armonk, NY: IBM Corp). The demographic variables of the respondents and results from part A of the questionnaire concerning the clinical handling of root resorption before publication in 2018 (T0) and after publication until 2021 (T1) were described using descriptive statistics, and the median and interquartile range (IQR) were calculated. The Mann–Whitney U test was used to determine differences between T0 and T1 and among the four domains. The Mann–Whitney U test was also used to determine median differences in cumulative scores for the degree of adherence to the guideline related to demographic variables. Multiple regression was used to examine the effect of the demographic variables (independent variables) on the degree of change in clinical practice towards the guideline recommendations from T0 to T1 (dependent variable). The sample size was calculated using G*Power 3.1 given an alpha of 0.05, a power of 0.8, and an effect size of 0.3, indicating a sample size of at least 94 respondents [12].

## Results

### Sample

In June 2021, e-mails containing a link to the REDCap questionnaire were sent out to 275 orthodontists. Initially, 58 orthodontists completed the questionnaire. Reminders via email and telephone increased the response to a total of 133, resulting in a response rate of 48%. One respondent was not clinically active at the time of completing the questionnaire and was therefore excluded. The inclusion period ended on January 7, 2022. The final study population consisted of 132 orthodontists. Table 1 shows the demographic characteristics of the study population.

**Table 1:** Demographics of the respondents (N = 132)

	Number of participants (%)
Sex	
Female	59 (45%)
Male	73 (55%)
Orthodontic education in	
The Netherlands	107 (81%)
Elsewhere	25 (19%)
Clinical experience	
0–5 years	15 (11%)
> 6 years	117 (89%)
Employment	
Private practice*	117 (89%)
Other**	15 (11%)

*Employment as practice owner or practice employee

**Working (part-time) in a hospital, university, or specialist centre

### Root resorption diagnostics

**Fig. 1:**
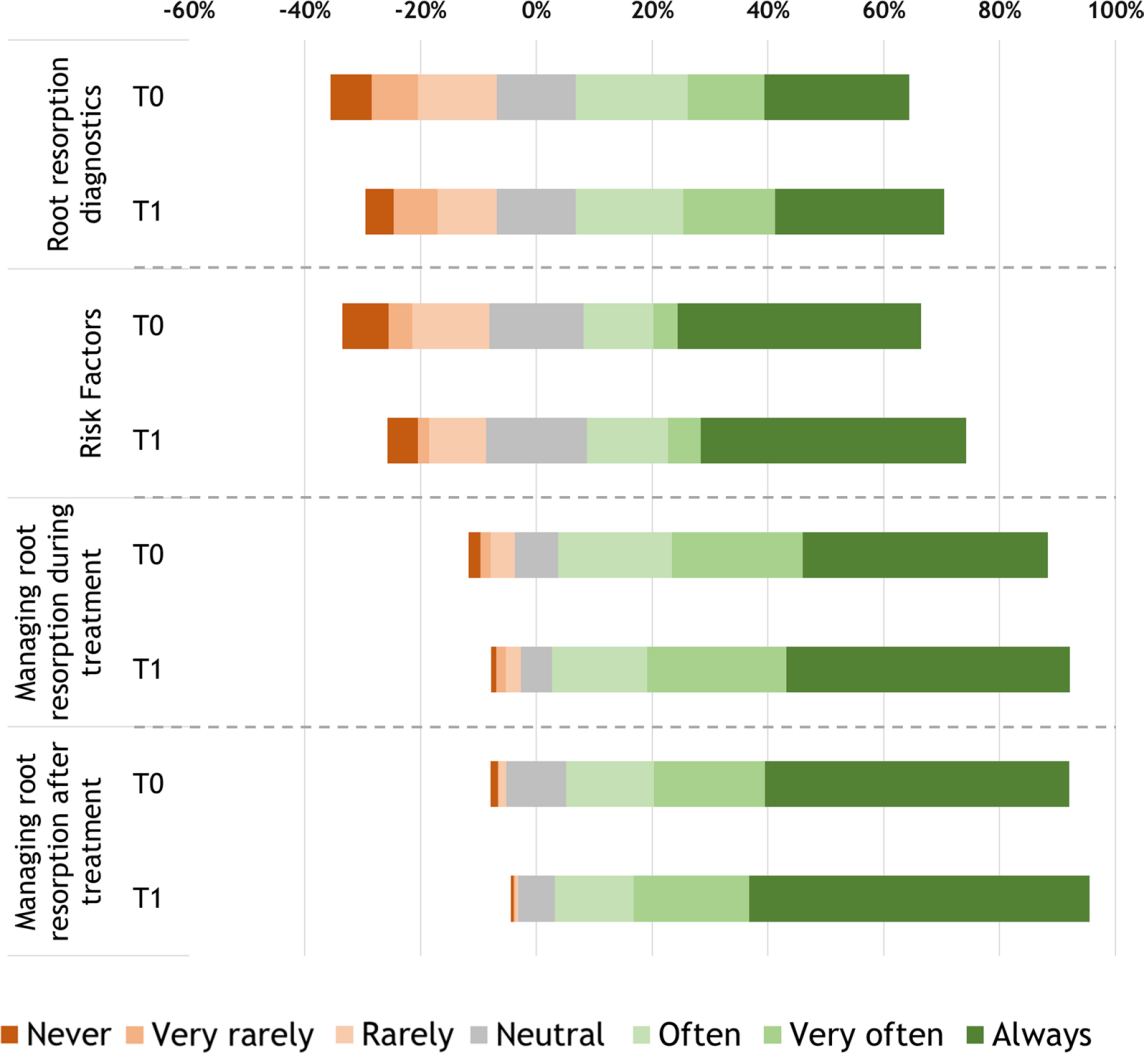
Compliance with the recommendations of the orthodontist guideline in the Netherlands before (T0) and 3 years after (T1) publication

Table 2 and Fig. 1 show the clinical practices regarding the EARR guideline recommendations before (T0) and after publication in 2018 (T1). Cumulatively, after publication of the guideline, most orthodontists (68.9%) did not act clinically different when diagnosing root resorption. However, 35 (26.5%) respondents positively changed their clinical behaviour, meaning their clinical practices changed towards the recommendations of the guideline, whereas 6 (4.6%) scored negatively between T0 and T1. In accordance with the guideline, in extraction therapy patients, a panoramic X-ray is very often (median = 6) obtained 12 months after the start of orthodontic treatment with fixed appliances, and 28 (21.2%) of the orthodontists changed positively in regard to their practices. Most orthodontists scored neutral (median = 4) regarding the consideration of making additional peri-apical radiographs if the already available radiographs do not provide sufficient information about the roots of the teeth, but 18 (13.7%) changed positively after introduction of the guideline; only 1 (0.7%) respondent scored negatively at T1.

**Table 2:** Clinical practice regarding the recommendations of the Orthodontic Root Resorption Guideline by orthodontists in the Netherlands before (T0) and after (T1) publication

	T0 Median (IQR)	T1 Median (IQR)	Positive difference T1-T0 (N)^a^	No difference T1–T0 (N)^b^	Negative difference T1–T0 (N)^c^	*P*-value T1-T0 (N)
*Root resorption* diagnostics*	10 (8–11)	10 (8–12)	35 (26.5%)	91 (68.9%)	6 (4.6%)	< 0.001
I consider making a panoramic X-ray 12 months after the start of the orthodontic treatment with fixed appliances in patients undergoing extraction therapy and compare this with a pre-treatment panoramic X-ray	6 (4–7)	6 (5–7)	28 (21.2%)	99 (75.0%)	5 (3.8%)	< 0.001
I consider taking additional peri-apical images if the already available X-rays do not provide enough information about the roots of the teeth	4 (3–5)	4 (3–6)	18 (13.7%)	113 (85.6%)	1 (0.7%)	0.001
Risk factors*	10 (8–11)	11 (9–12)	35 (26.5%)	96 (72.8%)	1 (0.7%)	< 0.001
I inform the patient about the risk of root resorption prior to orthodontic treatment	7 (5–7)	7 (6–7)	14 (10.7%)	118 (89.3%)	0	0.001
I inform the patient undergoing extraction therapy of the potential increased risk of developing more severe root resorption	4 (3–5)	4 (3–5)	31 (23.6%)	100 (75.7%)	1 (0.7%)	< 0.001
Treatment strategy if root resorption occurred during treatment*	35 (31–39)	37 (33–40)	53 (40.2%)	78 (59.1%)	1 (0.7%)	< 0.001
After the occurrence of apical root resorption (≥ 2 mm), I review the treatment goals and treatment plan and discuss the consequences, the patient's wishes, and the treatment goals with the patient	7 (5–7)	7 (6–7)	20 (15.2%)	112 (84.8%)	0	< 0.001
In case of severe generalised root resorption (≥ 4 mm root length loss), I consider discontinuing the treatment	6 (5–7)	6 (6–7)	14 (10.7%)	117 (88.6%)	1 (0.7%)	0.001
In the case of severe local root resorption (≥ 4 mm root length loss), I consider ending force application to the affected teeth	6 (5–7)	6 (6–7)	19 (14.5%)	112 (84.8%)	1 (0.7%)	< 0.001
If active treatment is continued, I consider a 3-month break before continuing treatment. During this interruption, the appliance must be made passive in such a way that the affected teeth are no longer loaded	5 (3–7)	6 (4–7)	35 (26.5%)	94 (71.2%)	3 (2.3%)	< 0.001
If the treatment is continued, I try to limit movement of the affected teeth as much as possible	6 (5–7)	7 (6–7)	16 (12.1%)	115 (87.2%)	1 (0.7%)	0.001
If active treatment is continued, I consider taking an X-ray of the affected teeth 6 months after restarting treatment	6 (5–7)	7 (5–7)	28 (21.2%)	102 (77.3%)	2 (1.5%)	< 0.001
What to do in patients with root resorption at the end of treatment*	19 (16–20)	19 (18–21)	37 (28.1%)	95 (71.9%)	0	< 0.001
I follow-up with the patient according to my regular retention protocol described in the ‘’Retention in Orthodontics’’ guideline (Wouters [13])	6 (5–7)	7 (6–7)	23 (17.4%)	109 (82.6%)	0	< 0.001
At the end of the orthodontic treatment, I ensure good communication with the patient about expectations regarding the affected tooth	7 (6–7)	7 (6–7)	16 (12.1%)	116 (87.9%)	0	< 0.001
I ensure good communication with the dentist at the end of the orthodontic treatment	7 (5–7)	7 (6–7)	19 (14.5%)	112 (84.8%)	1 (0.7%)	< 0.001
Cumulative score of the questionnaire**	74 (66–79)	83 (76–87)	130 (98.5%)	0	2 (1.5%)	< 0.001

Outcome measured on a scale of 1–7, where 1 = never, 2 = very rarely, 3 = rarely, 4 = neutral, 5 = often, 6 = very often, 7 = always

*Median and IQR calculated based on the total score of the recommendations in the domain

**Median and IQR calculated based on the total score of the entire questionnaire

^a^Number of respondents who scored higher at T1 than T0, indicating a change in clinical practice towards the guideline recommendations

^b^Number of respondents who scored equal at T1 as at T0, indicating no change in clinical practice towards the guideline recommendations

^c^Number of respondents who scored lower at T1 than T0, indicating a change in clinical practice in contrast to the guideline recommendations

### Risk factors

Orthodontists nearly always inform patients about the risk of root resorption (median = 7), and 14 (10.7%) orthodontists changed positively after introduction of the guideline. The question of providing information about a possibly increased risk of developing severe root resorption after extraction therapy was answered more neutrally (median = 4), but after publication of the guideline in 2018, 35 (26.5%) orthodontists changed their clinical practice according to the recommendations of the guideline.

### Treatment strategy if root resorption occurred during treatment

The largest change in clinical practice after publication of the guideline was seen within this domain. Cumulatively, 53 (40.2%) respondents scored positively between T0 and T1. After the occurrence of EARR of ≥ 2 mm, almost all orthodontists review the treatment plan and discuss the consequences, the patient’s wishes, and the treatment goals with the patient (median = 7). In cases of severe generalised root resorption (≥ 4 mm root length loss), most orthodontists consider discontinuing the treatment (median = 6). In addition, in cases of severe local root resorption (≥ 4 mm root length loss), most orthodontists consider not loading the affected teeth any more (median = 6). If active treatment is continued, a 3-month break is considered before continuing treatment. During this interruption, the appliance must be made passive in such a way that the affected teeth are no longer loaded, which was practised by most orthodontists at T1 (median = 6), and 35 (26.5%) respondents changed positively. If treatment is continued, most orthodontists try to limit movement of the affected teeth as much as possible (median = 7), the affected teeth are mostly (median = 7) moved as little as possible, and an X-ray is considered (median = 7) 6 months after restart. Twenty-eight (21.2%) orthodontists changed their practices positively.

### What to do in patients with root resorption at the end of treatment

Overall, 37 (28.1%) orthodontists changed their practices positively in this domain (median cumulative score = 19). After treatment, patients with root resorption are followed up according to the Dutch “Retention in Orthodontics” guideline (median = 7)[13]. At the end of treatment, expectations regarding the affected teeth are communicated with the patient (median = 7). There is also good communication with the dentist at the end of treatment regarding the patient’s root resorption (median = 7).

### Cumulative score of the questionnaire

The cumulative score in Table 2 and Fig. 1 shows that there has been a significant positive difference in the degree of follow-up of the entire guideline before and after publication in 2018 (T1-T0; p < 0.001). The maximum score that could be obtained was 91. The median total score for the entire guideline was 74 at T0 and 83 at T1, an increase of 9 points (12%).

### Influence of demographic variables

Table 3 shows the total scores for the different demographic variables. Multiple regression analysis showed that none of the demographic variables had a significant effect on the degree of change in clinical practice towards the guideline recommendations from T0 to T1 (data not shown).

**Table 3:** Cumulative scores for the degree of adherence to the guideline by demographic variable

	Number (%)	T0 Median (IQR)	T1 Median (IQR)	T1-T0^a^ Median (IQR)	*P*-value T1-T0	*P*-value Mann–Whitney U test
Female Male	59 (45%) 73 (55%)	74 (68–80) 73 (65–79)	82 (77–87) 83 (73–88)	7 (6–11) 7 (6–11)	< 0.001 < 0.001	0.994
Specialised in NL* Specialisation elsewhere	107 (81%) 25 (19%)	73 (67–79) 74 (66–80)	83 (77–87) 84 (71–89)	7 (6–11) 7 (5–10)	< 0.001 < 0.001	0.378
0–5 years experience ≥ 6 years experience	15 (11%) 117 (89%)	74 (66–80) 74 (66–80)	84 (71–89) 83 (77–88)	7 (5–10) 7 (6–11)	0.001 < 0.001	0.723
Private practice** Other***	117 (89%) 15 (11%)	73 (67–79) 77 (66–81)	83 (77–87) 85 (76–88)	7 (6–11) 7 (6–11)	< 0.001 0.001	0.428

*The Netherlands

**Employment as practice owner or practice employee

***Working (part-time) in a hospital, university, or specialist centre

^a^Sum score T1 – Sum score T0

## Discussion

This questionnaire assessed clinical practices regarding EARR 3 years after publication of a CPG, which was developed utilizing a rigorous and systematic approach [3, 8, 14, 15]. The findings demonstrated that, 3 years after introduction of the guideline, orthodontists improved their self-reported clinical practices to a more standardised management of root resorption. We found that 98.5% (N = 130) of the participating orthodontists changed their clinical practices towards the recommendations of the guideline. The largest difference was seen in the domain “Treatment strategy if root resorption is diagnosed during treatment”. The demographic characteristics of the respondents did not play a significant role in adherence to the guidelines.

### Study methodology and representativeness

This research consisted of a questionnaire asking the participants to reflect on their activities in the past and compare them to the present. A Likert-type scale is often used to investigate the adherence of individuals to a particular subject [16]. Respondents comprising a population that is more likely to respond in a nuanced way are better surveyed with a 7-point scale than with a 5-point scale [17]. Therefore, in the present study, we used a 7-point scale with the response items linked to a score of 1–7.

Previous studies demonstrated a higher response rate to digital questionnaires compared to written or telephone questionnaires [18]. Implementing pre-closure reminders has consistently yielded favorable outcomes in previous investigations, resulting in an average response rate increase of 12–20% [19]. This current study aligns with these findings. Through email and telephone reminders, the response rate experienced a substantial increase of 27%, escalating from an initial 58–133 participants. This surge elevated the overall response rate to 48%. Gender distribution within the study group was characterized by 45% females and 55% males. Moreover, 81% of orthodontists received their education in the Netherlands, while the remaining 19% obtained their training abroad. A reference to the database of the Royal Dutch Dental Association (KNMT) reveals that, as of September 2021, orthodontists in the Netherlands aged ≤ 67 years constituted 44% females and 56% males [20]. The geographical distribution of specialist education also mirrored that of the current research cohort, with 19% educated internationally and 81% within the Netherlands. This substantiates the representativeness of our research group to the target population. Although earlier studies have indicated a tendency for women and younger dentists to be more engaged in preventive measures and contemporary advancements [21], these patterns were not corroborated by our present findings. None of the demographic variables exhibited a discernible influence on clinical practices concerning the implementation of guideline recommendations.

To our knowledge this study on the effect of a clinical practice guideline for EARR is the first of its kind in the literature. Therefore we are not able to compare our results with other studies. Compared to orthodontists in many other countries, Dutch orthodontists demonstrate a high level of awareness regarding published CPGs. The implementation of CPGs in clinical practice is an essential part of the mandatory 5-year re-registration process for orthodontic specialists in the Netherlands. This accomplishment is further reinforced through the regular introduction and extensive discussions of guidelines during meetings of the Dutch Association of Orthodontists, their prominent inclusion in newsletters, and the significant importance attributed to CPGs within the professional discourse among Dutch orthodontists.

The guideline is based on evidence from the literature and expert opinion from consensus meetings of the committee consisting of Dutch orthodontists [22]. Therefore, guidelines may differ between countries. Most respondents (81%) were trained in the Netherlands, but the orthodontists trained elsewhere appeared to follow the recommendations to a similar extent. Furthermore, present employment status had no significant impact on implementation of the guideline. Thus, clinical practices geared towards the guideline are comparable for the entire population of orthodontists in the Netherlands.

### Domain responses

For the diagnosis of root resorption, the guideline recommends a panoramic X-ray 12 months after starting the orthodontic treatment with fixed appliances in patients undergoing extraction therapy. As mentioned previously, extraction of premolars in the context of orthodontic treatment with fixed appliances is the only risk factor for which evidence was found for an association with the severity of root resorption [3, 22, 23]. Therefore, a panoramic X-ray is recommended for patients undergoing extraction therapy 12 months after the start of orthodontic treatment with fixed appliances [3]. The respondents very often followed this recommendation.

To prevent severe EARR, the orthodontist should assess the risk in the individual patient prior to treatment. EARR is part of the core set of risks identified for discussion with patients as part of consent for orthodontic treatment [24]. The systematic literature search carried out in the context of the development of the guideline revealed low evidence for possible risk factors. Most orthodontists (86%) agree that root resorption should never go undiscovered during treatment [25]. Orthodontists participating in our study indicated that they always inform patients about the risk of root resorption. However, they appear to be more neutral towards informing patients about the increased risk of EARR in extraction therapy. Because of the importance of EARR, it is paramount to inform patients in advance about the risk of developing EARR. This is consistent with the findings in the present study.

In the guideline domain “When root resorption occurred during treatment”, 40.2% of the orthodontists positively changed their clinical practice, making it the domain with the greatest change. This shows that this domain was probably the most uncertain among orthodontists. After publication of the guideline, more than 20% of the orthodontists changed their clinical practices regarding question 8, ‘’a 3-month treatment break is considered in which the appliance is made passive’’ [4, 26], and question 10, ‘’after 6 months additional X-rays are considered to monitor the root resorption’’[27, 28]. Apparently, the guideline supported clinical practices, which may also reduce practice variation.

### Limitations

Participants were asked to reflect upon their past clinical activities and compare them with their current practices. Consequently, when interpreting the results of this study it is important to acknowledge that memory effects could potentially have influenced their recollections and assessments.

The main objective of the guideline is to improve and standardise the clinical procedures for orthodontic practices. This questionnaire addresses the clinical activities of orthodontists, but it remains unknown as to which strategies were used to clinically implement the guideline in daily practice. To overcome this limitation, conducting a qualitative study that explores the barriers and experiences of clinicians regarding the utilization of the presented CPG could provide valuable insights for the implementation of future guidelines. In addition, this study was limited to orthodontists only, the intended users of the guideline. Therefore, the results do not provide insight into the actions of general dentists, who perform a significant number of orthodontic treatments.

## Conclusion

Three years after introduction of the guideline, orthodontists demonstrated a change in clinical practice towards the recommendations, which resulted in a more standardised practice regarding root resorption. Results were not influenced by demographic predictors, proving that the guideline has been accepted among the Dutch orthodontics profession.

### Supplementary Information

**Additional file 1:** Questionnaire translated in English for publication purposes.

**Additional file 2:** Raw data file extracted from the UMCG REDCap datamanager.

## Data Availability

All data generated or analysed during this study are included in this published article [and its additional information files].

## Abbreviations

AGREE II instrument: Appraisal of Guidelines for Research and Evaluation II
BIG register: Dutch dental register
CPG: Clinical practice guideline
EARR: External apical root resorption
KNMT: Royal Dutch Dental Association
METc UMCG: Medical Ethics Review Board of the University Medical Center Groningen)
NVvO: Dutch Association of Orthodontists
REDCap: Research Electronic Data Capture
WMO: Medical Research Involving Human Subjects Act

## References

[1] Yassir YA, McIntyre GT, Bearn DR (2021). Orthodontic treatment and root resorption: an overview of systematic reviews. Eur J Orthod.

[2] Sameshima GT, Iglesias-Linares A (2021). Orthodontic root resorption. J World Fed Orthod.

[3] Sondeijker CFW, Lamberts AA, Beckmann SH, Kuitert RB, van Westing K, Persoon S, Kuijpers-Jagtman AM (2020). Development of a clinical practice guideline for orthodontically induced external apical root resorption. Eur J Orthod.

[4] Weltman B, Vig KW, Fields HW, Shanker S, Kaizar EE (2010). Root resorption associated with orthodontic tooth movement: a systematic review. Am J Orthod Dentofacial Orthop.

[5] Maués CP, do Nascimento RR, Vilella Ode V (2015). Severe root resorption resulting from orthodontic treatment: prevalence and risk factors. Dent Press J Orthod..

[6] Dos Santos CCO, Bellini-Pereira SA, Medina MCG, Normando D (2021). Allergies/asthma and root resorption: a systematic review. Prog Orthod.

[7] Lugtenberg M, Burgers JS, Westert GP (2009). Effects of evidence-based clinical practice guidelines on quality of care: a systematic review. Qual Saf Health Care.

[8] Schünemann H, Brożek J, Guyatt G, Oxman A, eds. GRADE Handbook for Grading Quality of Evidence and Strength of Recommendations. Grade Working Group. Published 2013. Accessed June 19, 2023. https://gradepro.org/

[9] Burns KE, Duffett M, Kho ME, Meade MO, Adhikari NK, Sinuff T (2008). A guide for the design and conduct of self-administered surveys of clinicians. CMAJ.

[10] Harris PA, Taylor R, Thielke R, Payne J, Gonzalez N, Conde JG (2009). Research electronic data capture (REDCap)–a metadata-driven methodology and workflow process for providing translational research informatics support. J Biomed Inform.

[11] Harris PA, Taylor R, Minor BL, Elliott V, Fernandez M, O'Neal L (2019). The RED Cap consortium: building an international community of software platform partners. J Biomed Inform.

[12] Faul F, Erdfelder E, Lang AG, Buchner A (2007). G*Power 3: a flexible statistical power analysis program for the social, behavioral, and biomedical sciences. Behav Res Methods.

[13] Wouters C, Lamberts TA, Kuijpers-Jagtman AM, Renkema AM (2019). Development of a clinical practice guideline for orthodontic retention. Orthod Craniofac Res.

[14] Brouwers MC, Kho ME, Browman GP, Burgers JS, Cluzeau F, Feder G (2010). AGREE Next Steps Consortium. AGREE II: advancing guideline development, reporting and evaluation in health care. CMAJ.

[15] Kwaliteitsinstituut voor de Gezondheidszorg CBO (Centraal Begeleidingsorgaan). Evidence-based Richtlijnontwikkeling, Handleiding voor werkgroepleden Kennisbank richtlijnontwikkeling. Accessed February 1, 2023. https://www.huidziekten.nl/diversen/opleiding/CATDatabase/EBROhandleidingrichtlijnontwikkeling.pdf

[16] Barua A. Methods for Decision-Making in Survey Questionnaires Based on Likert Scale. J Asian Sci Res. 2013;3(1):35–38. https://archive.aessweb.com/index.php/5003/article/view/3446

[17] Dawes J (2008). Do data characteristics change according to the number of scale points used? An experiment using 5-point, 7-point and 10-point scales. Int J Market Res.

[18] Renkema AM, Sips ET, Bronkhorst E, Kuijpers-Jagtman AM (2009). A survey on orthodontic retention procedures in The Netherlands. Eur J Orthod.

[19] Meyer VM, Benjamens S, Moumni ME, Lange JFM, Pol RA (2022). Global overview of response rates in patient and health care professional surveys in surgery: a systematic review. Ann Surg.

[20] KNMT-afdeling Onderzoek. Tandartsen in Nederland: wie, wat en waar [Dentists in the Netherlands: who, what, and where]. NT Dentz. 2021(Sep):16–18.

[21] Yusuf H, Tsakos G, Ntouva A, Murphy M, Porter J, Newton T (2015). Differences by age and sex in general dental practitioners' knowledge, attitudes and behaviours in delivering prevention. Br Dent J.

[22] Kuijpers-Jagtman AM, Sondeijker CFW, Beckmann SH, Kuitert RB, van Westing K. Wortelresorptie in de orthodontie. NVvO. Published 2018. Accessed February 1, 2023. https://orthodontist.nl/pdf/wortelresorptie-in-de-orthodontie.pdf

[23] Artun J, Van’t Hullenaar R, Doppel D, Kuijpers-Jagtman AM (2009). Identification of orthodontic patients at risk of severe apical root resorption. Am J Orthod Dentofacial Orthop..

[24] Perry J, Popat H, Johnson I, Farnell D, Morgan MZ (2021). Professional consensus on orthodontic risks: what orthodontists should tell their patients. Am J Orthod Dentofacial Orthop.

[25] Jerrold L, Danoff-Rudick J (2022). Never events in clinical orthodontic practice. Am J Orthod Dentofacial Orthop.

[26] Levander E, Malmgren O, Eliasson S (1994). Evaluation of root resorption in relation to two orthodontic treatment regimes. A clinical experimental study. Eur J Orthod..

[27] Artun J, Smale I, Behbehani F, Doppel D, Van't Hof M, Kuijpers-Jagtman AM (2005). Apical root resorption six and 12 months after initiation of fixed orthodontic appliance therapy. Angle Orthod.

[28] Smale I, Artun J, Behbehani F, Doppel D, van’t Hof M, Kuijpers-Jagtman AM (2005). Apical root resorption 6 months after initiation of fixed orthodontic appliance therapy. Am J Orthod Dentofacial Orthop.

