# Supplementary material for: Evaluation of the use of a clinical practice guideline for external apical root resorption among orthodontists
Source: Prog Orthod. 2024 Apr 22;25:15. doi: 10.1186/s40510-024-00515-5 (PMC11033249; doi:10.1186/s40510-024-00515-5)
Supplement: Supplementary file 1 — Additional file 1: Questionnaire translated in English for publication purposes. [file 40510_2024_515_MOESM1_ESM.docx]

**Questionnaire to evaluate the use of the clinical practice guideline for external apical root resorption in the Netherlands**

**Introduction**

In 2018, the Dutch Association of Orthodontists (NVvO) in collaboration with the Knowledge Institute of the Federation of Medical Specialists (KIMS) developed and published the guideline "Root Resorption in Orthodontics." This guideline provides an overview of the current scientific knowledge regarding apical root resorption and offers evidence-based recommendations for clinical practice.

The purpose of this study is to investigate the extent to which orthodontists in the Netherlands have followed the recommendations and implemented the guideline, now that it has been in use for three years. The first part of the questionnaire presents a series of recommendations from the guideline, followed by questions for each recommendation regarding how you clinically managed apical root resorption before the publication of the guideline in 2018 and from publication until now in 2021. The second part consists of five general questions.

Completing the questionnaire will take approximately 5-10 minutes.

Thank you very much for your cooperation!

**Informed consent**

I declare that I have been clearly informed of the nature and methodology of this study, as described in the introduction to this questionnaire. By completing and submitting the questionnaire, I consent for my data being used anonymously for research purposes as explained in the introductory email. I understand that my participation in this research is voluntary.

'My data will be treated confidentially and will only be used for this study.

If you would like to have more information, before giving your consent or in the future, please contact xx (Department of Orthodontics) via the email address: ….

I understand the above text and agree to participate in the study.

- Yes

**PART A: QUESTIONS REGARDING THE GUIDELINE**

**Root resorption diagnostics**

1. I consider making a panoramic X-ray 12 months after the start of the orthodontic treatment with fixed appliances in patients undergoing extraction therapy and compare this with a pre-treatment panoramic X-ray.

Before the guideline publication in 2018:

- Never
- Very rarely
- Rarely
- Neutral
- Often
- Very often
- Always

After the publication of the guideline in 2018 so far:

- Never
- Very rarely
- Rarely
- Neutral
- Often
- Very often
- Always

**Root resorption diagnostics**

2. I consider taking additional peri-apical images if the already available X-rays do not provide enough information about the roots of the teeth.

Before the guideline publication in 2018:

- Never
- Very rarely
- Rarely
- Neutral
- Often
- Very often
- Always

After the publication of the guideline in 2018 so far:

- Never
- Very rarely
- Rarely
- Neutral
- Often
- Very often
- Always

**Risk factors**

3. I inform the patient about the risk of root resorption prior to orthodontic treatment.

Before the guideline publication in 2018:

- Never
- Very rarely
- Rarely
- Neutral
- Often
- Very often
- Always

After the publication of the guideline in 2018 so far:

- Never
- Very rarely
- Rarely
- Neutral
- Often
- Very often
- Always

**Risk factors**

4. I inform the patient undergoing extraction therapy of the potential increased risk of developing more severe root resorption.

Before the guideline publication in 2018:

- Never
- Very rarely
- Rarely
- Neutral
- Often
- Very often
- Always

After the publication of the guideline in 2018 so far:

- Never
- Very rarely
- Rarely
- Neutral
- Often
- Very often
- Always

**Treatment strategy if root resorption occurred during treatment**

5. After the occurrence of apical root resorption (≥ 2 mm), I review the treatment goals and treatment plan and discuss the consequences, the patient's wishes, and the treatment goals with the patient.

Before the guideline publication in 2018:

- Never
- Very rarely
- Rarely
- Neutral
- Often
- Very often
- Always

After the publication of the guideline in 2018 so far:

- Never
- Very rarely
- Rarely
- Neutral
- Often
- Very often
- Always

**Treatment strategy if root resorption occurred during treatment**

6. In case of severe generalised root resorption (≥ 4 mm root length loss), I consider discontinuing the treatment.

Before the guideline publication in 2018:

- Never
- Very rarely
- Rarely
- Neutral
- Often
- Very often
- Always

After the publication of the guideline in 2018 so far:

- Never
- Very rarely
- Rarely
- Neutral
- Often
- Very often
- Always

**Treatment strategy if root resorption occurred during treatment**

7. In the case of severe local root resorption (≥ 4 mm root length loss), I consider ending force application to the affected teeth

Before the guideline publication in 2018:

- Never
- Very rarely
- Rarely
- Neutral
- Often
- Very often
- Always

After the publication of the guideline in 2018 so far:

- Never
- Very rarely
- Rarely
- Neutral
- Often
- Very often
- Always

**Treatment strategy if root resorption occurred during treatment**

8. If active treatment is continued, I consider a 3-month break before continuing treatment. During this interruption, the appliance must be made passive in such a way that the affected teeth are no longer loaded.

Before the guideline publication in 2018:

- Never
- Very rarely
- Rarely
- Neutral
- Often
- Very often
- Always

After the publication of the guideline in 2018 so far:

- Never
- Very rarely
- Rarely
- Neutral
- Often
- Very often
- Always

**Treatment strategy if root resorption occurred during treatment**

9. If the treatment is continued, I try to limit movement of the affected teeth as much as possible.

Before the guideline publication in 2018:

- Never
- Very rarely
- Rarely
- Neutral
- Often
- Very often
- Always

After the publication of the guideline in 2018 so far:

- Never
- Very rarely
- Rarely
- Neutral
- Often
- Very often
- Always

**Treatment strategy if root resorption occurred during treatment**

10. If active treatment is continued, I consider taking an X-ray of the affected teeth 6 months after restarting treatment.

Before the guideline publication in 2018:

- Never
- Very rarely
- Rarely
- Neutral
- Often
- Very often
- Always

After the publication of the guideline in 2018 so far:

- Never
- Very rarely
- Rarely
- Neutral
- Often
- Very often
- Always

**What to do in patients with root resorption at the end of treatment**

11. I follow-up with the patient according to my regular retention protocol described in the ‘’Retention in Orthodontics’’ guideline (Wouters 2019).

Guideline Retention in Orthodontics

[Attachment: "Recommendations guideline Retention in orthodontics.pdf"]

Before the guideline publication in 2018:

- Never
- Very rarely
- Rarely
- Neutral
- Often
- Very often
- Always

After the publication of the guideline in 2018 so far:

- Never
- Very rarely
- Rarely
- Neutral
- Often
- Very often
- Always

**What to do in patients with root resorption at the end of treatment**

12. At the end of the orthodontic treatment, I ensure good communication with the patient about expectations regarding the affected tooth.

Before the guideline publication in 2018:

- Never
- Very rarely
- Rarely
- Neutral
- Often
- Very often
- Always

After the publication of the guideline in 2018 so far:

- Never
- Very rarely
- Rarely
- Neutral
- Often
- Very often
- Always

**What to do in patients with root resorption at the end of treatment**

13. I ensure good communication with the dentist at the end of the orthodontic treatment.

Before the guideline publication in 2018:

- Never
- Very rarely
- Rarely
- Neutral
- Often
- Very often
- Always

After the publication of the guideline in 2018 so far:

- Never
- Very rarely
- Rarely
- Neutral
- Often
- Very often
- Always

**PART B: GENERAL QUESTIONS**

1.What is your gender?

- Male
- Female
- Other

2a. Where were you trained as an orthodontist?

- The Netherlands
- Elsewhere

3. How many years of clinical experience do you have after training as an orthodontist?

- 0-5 years
- >5 years

4. Present employment (multiple answers possible):

- As a practice owner
- As a practice employee, not a practice owner
- In a hospital
- At a university
- In a specialist centre
- Not practicing
- Otherwise, please explain:

Is there anything else you would like to share that has not been addressed in this questionnaire?
